# Supplementary material for: Interoception of breathing and its relationship with anxiety
Source: Neuron. 2021 Dec 15;109(24):4080–4093.e8. doi: 10.1016/j.neuron.2021.09.045 (PMC8691949; doi:10.1016/j.neuron.2021.09.045)
Supplement: Document S1. Figures S1–S7 and Tables S1–S7 [file mmc1.pdf]

Neuron, Volume *109*

Supplemental information

Interoception of breathing

and its relationship with anxiety

Olivia K. Harrison, Laura Köchli, Stephanie Marino, Roger Luechinger, Franciszek Hennel, Katja Brand, Alexander J. Hess, Stefan Frässle, Sandra Iglesias, Fabien Vinckier, Frederike H. Petzschner, Samuel J. Harrison, and Klaas E. Stephan

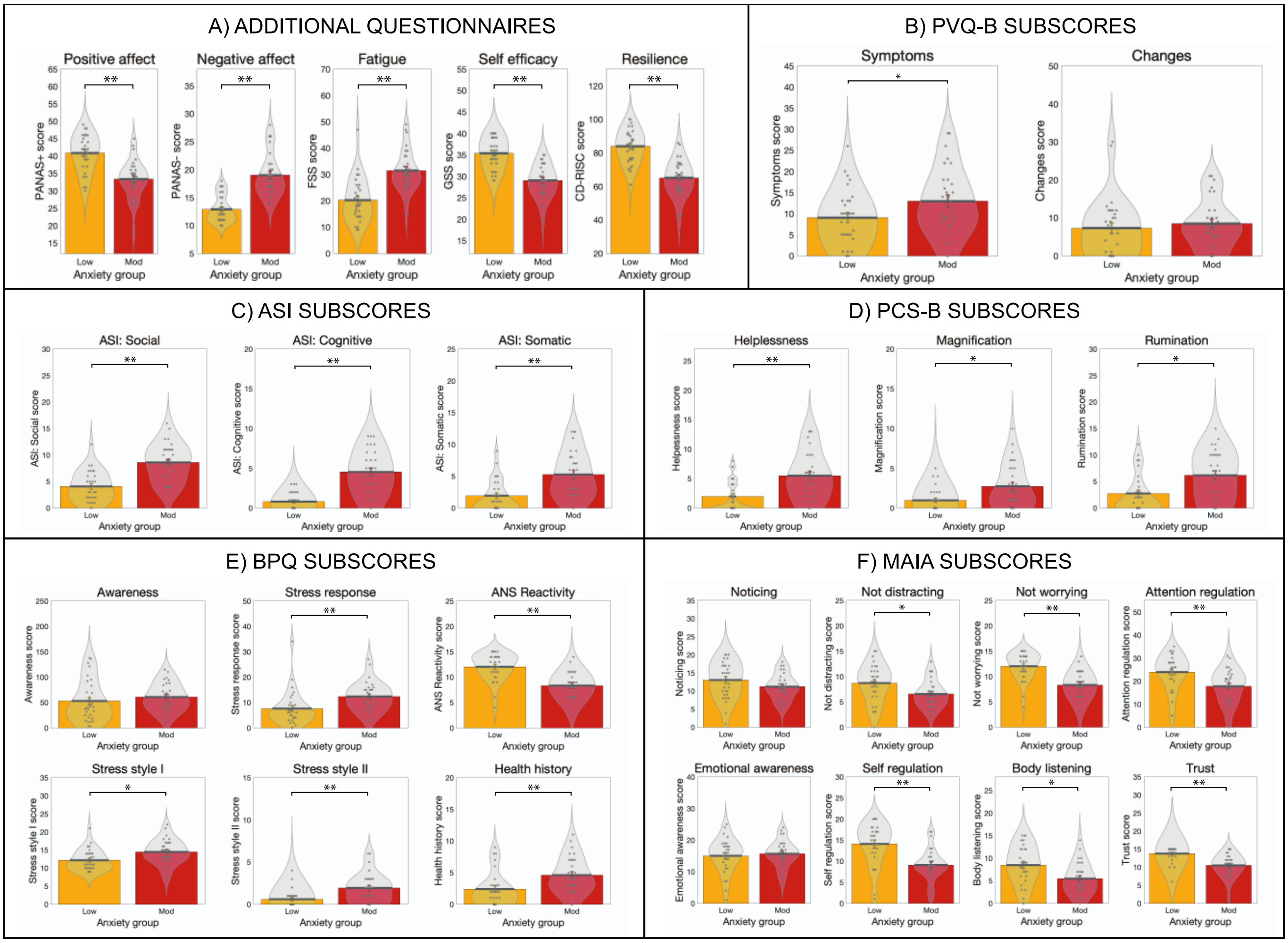

*Supplementary Figure 1. Results from the additional questionnaires measured in groups of healthy individuals with either low or moderate levels of anxiety, related to Figure 1. Participants with low anxiety scored 20-25 on the Spielberger Trait Anxiety Inventory (STAI-T), and those with moderate anxiety scored 35+ on the STAI-T. Questionnaires: A) 'Positive affect' and 'Negative affect' from the Positive Affect Negative Affect Schedule (PANAS-T), 'Fatigue' from the Fatigue Severity Scale (FSS), 'Self efficacy' from the General Self-Efficacy Scale, and 'Resilience' from the Connor-Davidson Resilience Scale; B) Sub-scores of the Pain Vigilance Awareness Questionnaire (with the word 'pain' substituted for 'breathing', PVQ-B); C) Sub-scores of the Anxiety Sensitivity Index (ASI-3) questionnaire; D) Sub-scores of the Pain Catastrophising Scale (with the word 'pain' substituted for 'breathing', PCS-B); E) Sub-scores of the Body Perception Questionnaire (BPQ); F) Sub-scores of the Multidimensional Assessment of Interoceptive Awareness Questionnaire (MAIA). Bar plots represent mean±standard error values, with the distribution of values overlaid in grey. **Significant at p<0.01, with no correction for multiple comparisons (exploratory results). Bar plots represent mean±standard error values, with the distribution of values overlaid in grey. Bar plot code adapted from the CANLAB Toolbox (https://github.com/canlab).*

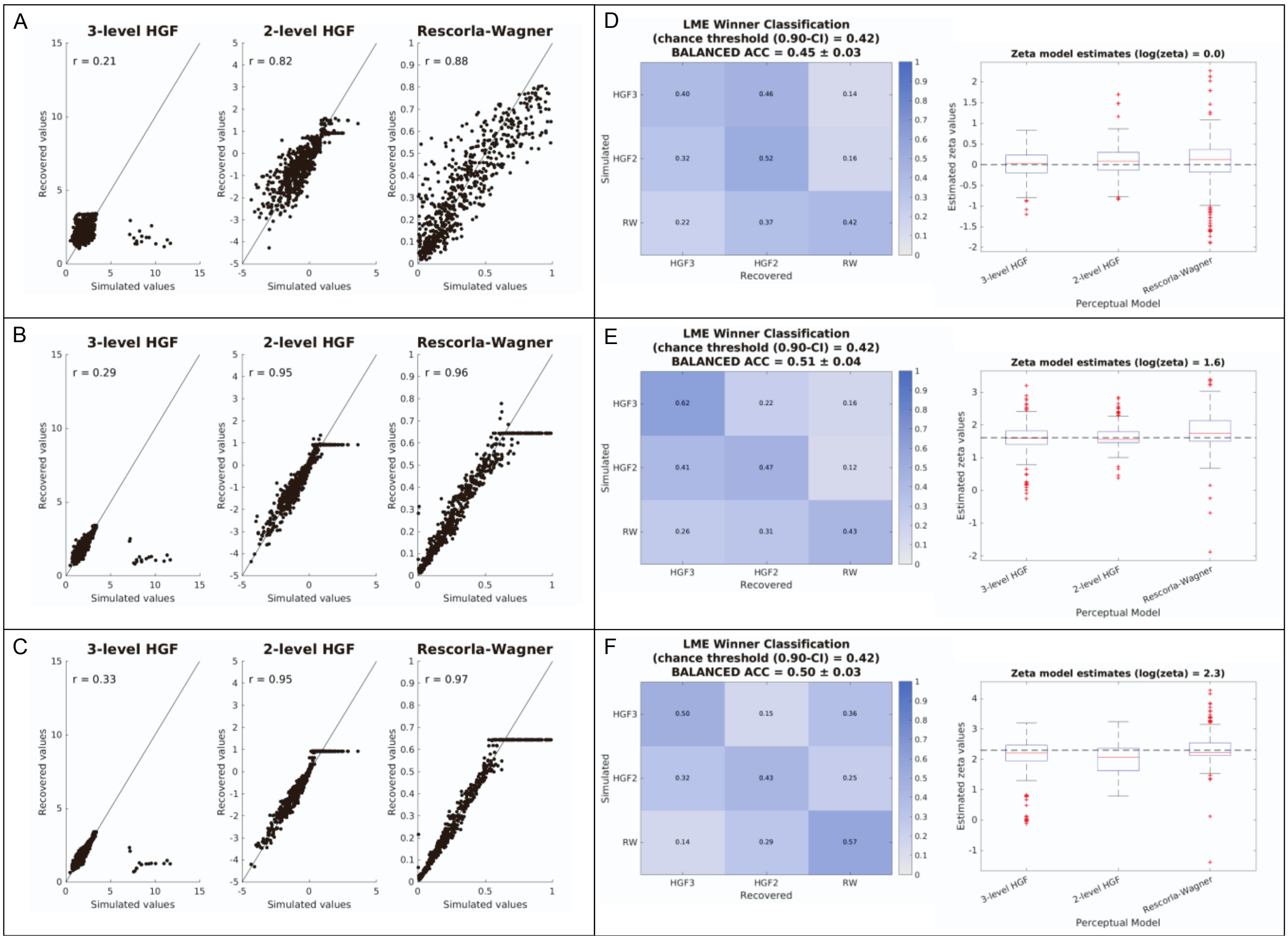

Supplementary Figure 2. Parameter recovery and model identifiability for the three candidate models, related to Figure 3. A-C) Demonstration of parameter recovery using simulated participants from the prior distributions presented in Supplementary Table 2. 60 simulated participant responses were generated using 10 different seed values, totalling n=600 simulations plotted here. D-E) Demonstration of model identifiability using simulated participants from the prior distributions presented in Supplementary Table 2. 60 simulated participant responses were generated using 10 different seed values, and the confusion matrices, balanced accuracy and zeta estimates are the average values across the 10 simulation runs. Three noise levels were used for the simulations, with an inverse decision temperature (ζ) of 1 (A,D), 5 (B,E) and 10 (C,E), representing very noisy ($\zeta = 1$) *to very deterministic* ($\zeta = 10$) *settings.*

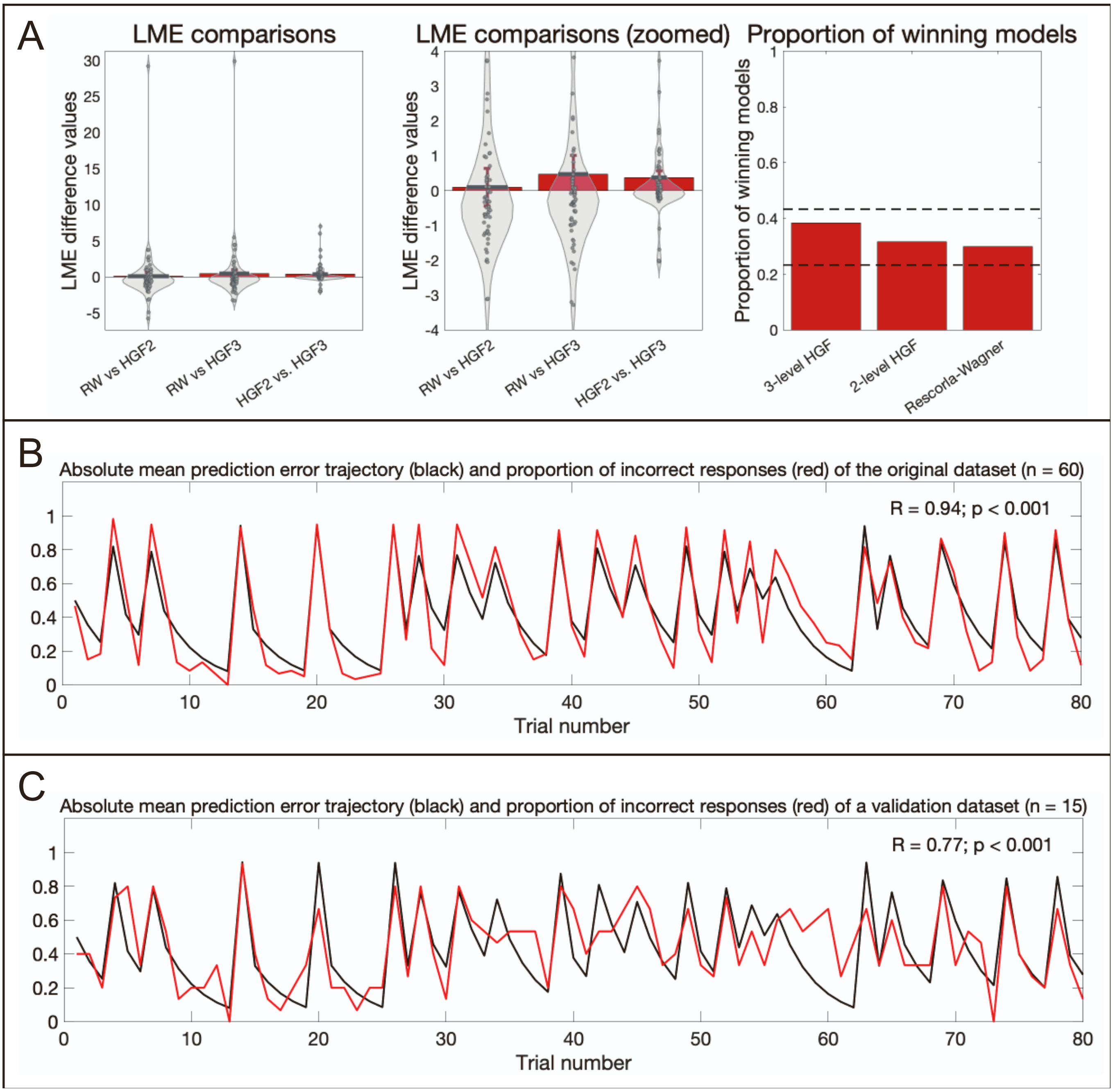

Supplementary Figure 3. Model comparisons and fit quality of the chosen model (the Rescorla Wagner), related to Figure 3. A) Model comparison: Estimated log model evidence (LME) values across all participants. Left and middle: Comparisons between each model pair, where bar plots represent mean±standard error values of the specified differences in LME, with the distribution of values overlaid in grey. Bar plot code adapted from the CANLAB Toolbox (https://github.com/canlab). Right: Proportion of highest LME values across all 60 participants. Dotted lines represent upper and lower 95% confidence intervals for chance, and all proportions of winner classifications lie within the chance range. B) Model validation in original data: Comparison of the mean prediction error trajectory (black) against the proportion of participants giving incorrect responses at each trial (red) for the original dataset (n = 60). The close correlation between these trajectories demonstrates the extent to which the chosen model (the Rescorla Wagner) captures important aspects of participant performance. C) Model validation in unseen data: Comparison of the mean prediction error trajectory from the original dataset (black) against the proportion of participants giving incorrect responses at each trial (red) for a validation dataset (n = 15), who were not preselected for any specific anxiety level. The correlation between these trajectories demonstrates the extent to which the average model fit for the original data is able to capture important aspects of participant performance on ***unseen*** *data. The prediction error trace in (B) and (C) is the absolute prediction error trajectory from the participant with the closest learning rate to the mean across all participant model fits. The prediction error trace is represented in stimulus space (see Supplementary Figure 4 for transformation procedures).*

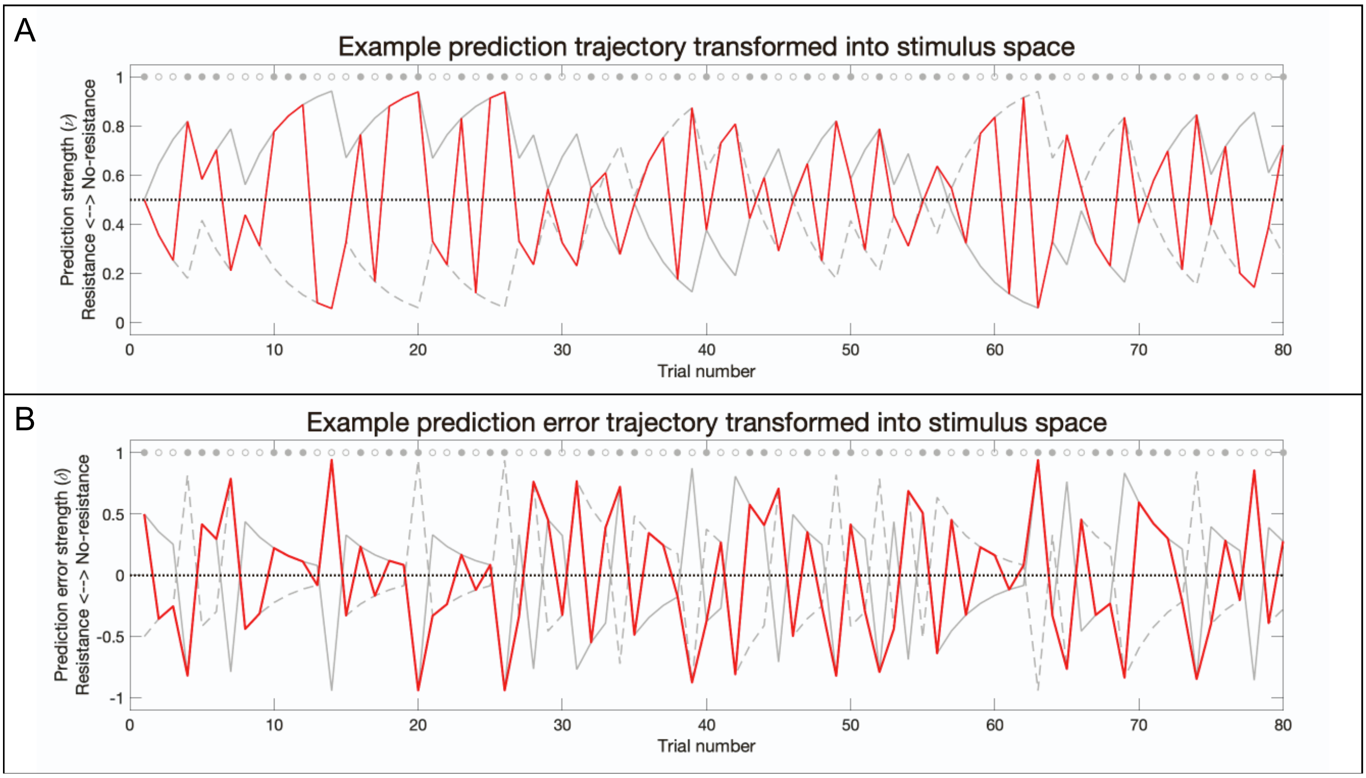

Supplementary Figure 4. Transformation of the prediction and prediction error trajectories from contingency space to stimulus space, related to Figures 4 and 5. A) The fitted trajectory (in contingency space) is demonstrated by the solid grey line, where the value 1 is assigned when one cue (cue 1) predicts no resistance and the opposing cue (cue 2) predicts a resistance (the value 0 is assigned for the opposing conditions). The trajectories were then transformed into stimulus space, where a value of 1 was assigned when no resistance was delivered, while a value of 0 was assigned when a resistance was delivered. For this transformation, a mirrored trajectory was firstly generated (dashed grey line) to represent the second cue, as the participants were explicitly told that the cues acted as a pair that had opposite probabilities (20% or 80%) of predicting resistance. The solid grey trajectory thus represents the cue that started with an 80% probability of being followed by no resistance in stimulus space (cue 1), while the dashed grey line represents the cue that started with a 20% probability of being followed by no resistance (cue 2). The values at each trial were taken from the trajectory of the cue that was presented at that trial: either cue 1 (trials with a closed grey circle) or cue 2 (trials with an open grey circle). The same transformation was performed on the prediction error trajectories in (B), where the solid grey line represents the prediction error associated with cue 1, while the dashed grey line represents the prediction error associated with cue 2. The example trajectories were taken from the participant with the closest learning rate to the mean value across all participants.

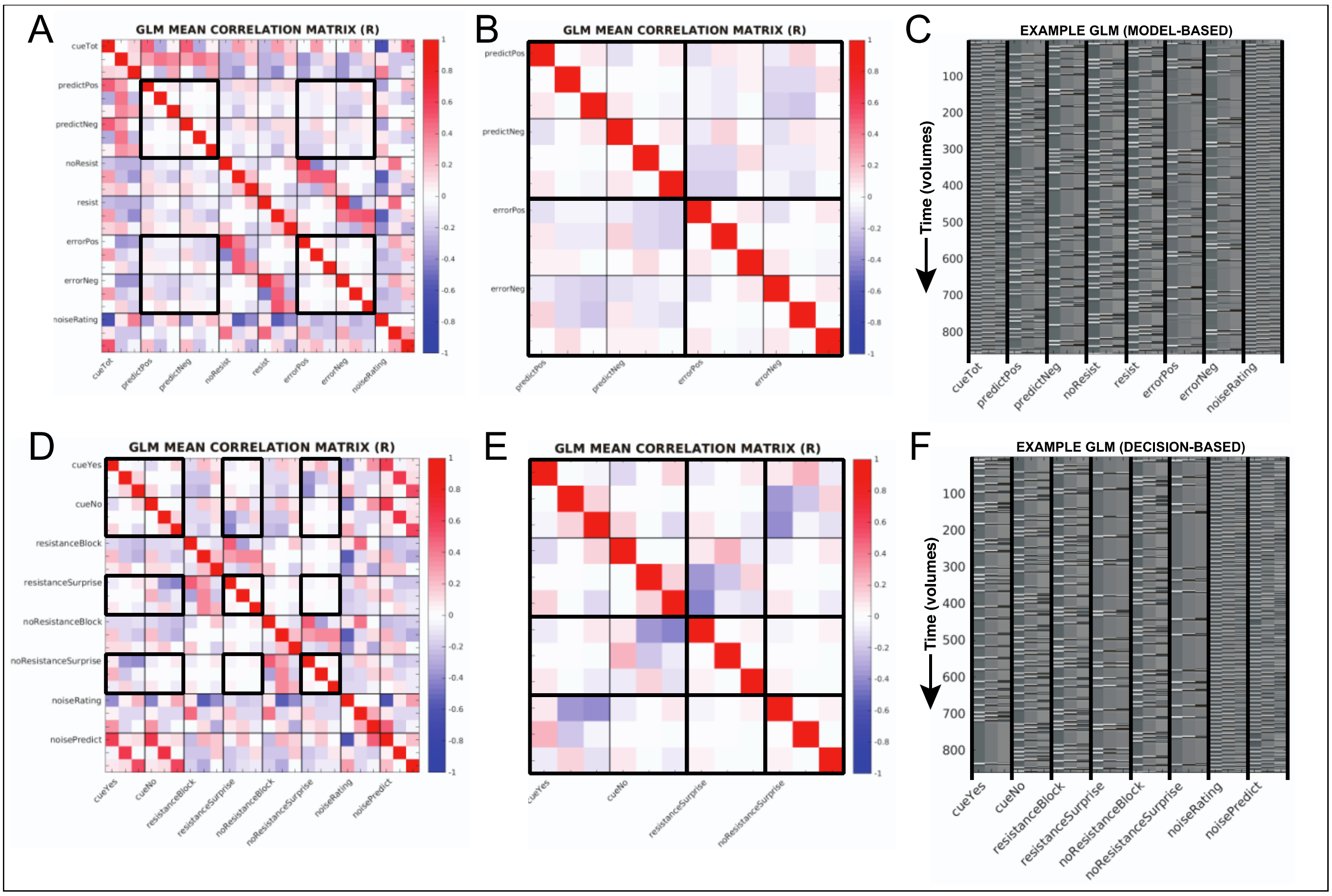

Supplementary Figure 5. Demonstration of the correlation between regressors for the model-based and decision-based general linear models, related to Figure 4 and 5. A and D) Average correlation matrices (using Fischer's R-to-Z transformation prior to averaging) from all single subject general linear models used in the model-based (A) and decision-based (D) fMRI analyses (noise regressors not shown). B and E) Targeted correlation matrices to demonstrate the relationship between predictions and errors for A and D, respectively. C) An example general linear model from a single participant model-based fMRI analysis. Each of the main regressors also include a temporal and dispersion derivative. Please see STAR methods for a full description of each regressor. F) An example general linear model from a single participant decision-based fMRI analysis. Each of the main regressors also include a temporal and dispersion derivative, and consist of: 1) 'cueYes' – the time periods covering the presentation of cues where the participant predicted an upcoming resistance; 2) 'cueNo' – the time periods covering the presentation of cues where the participant predicted no upcoming resistance; 3) 'resistanceBlock' – the stimulus periods when resistance was applied, from the onset of the inspiration against the increased inspiratory pressure following the presentation of the circle cue to the end of the circle presentation; 4) 'resistanceSurprise' – resistance stimuli that were surprising (i.e. when participant had predicted no resistance), with an onset at the beginning of the corresponding stimulus period and a duration of 0.5 seconds; 5) 'noResistanceBlock' – the stimulus periods when no resistance was applied, from the onset of the first inspiration following the presentation of the circle cue to the end of the circle presentation; 6) 'noResistanceSurprise' – no-resistance stimuli that were surprising (i.e. when participant had predicted resistance), with an onset at the beginning of the corresponding stimulus period and a duration of 0.5 seconds; 7) 'noiseRating' – the periods at the end of each trial where the participant rated the intensity of the previous stimulus, with an onset at the beginning and duration that encompassed the length of the rating period. 8) 'noisePredict' – the button press periods during the cue presentation, with an onset given by the response time for the button press on each trial and a duration of 0.5 seconds. Noise regressors not shown: the convolved end-tidal carbon dioxide trace (plus temporal and dispersion derivatives), the RETROICOR and convolved respiratory volume per unit of time (RVT) regressors provided by the PhysIO toolbox, 6 motion regressors and 6 extended motion regressors (derivatives), and the timeseries of all identified noise components from the independent component analysis conducted during preprocessing were also included in the model.

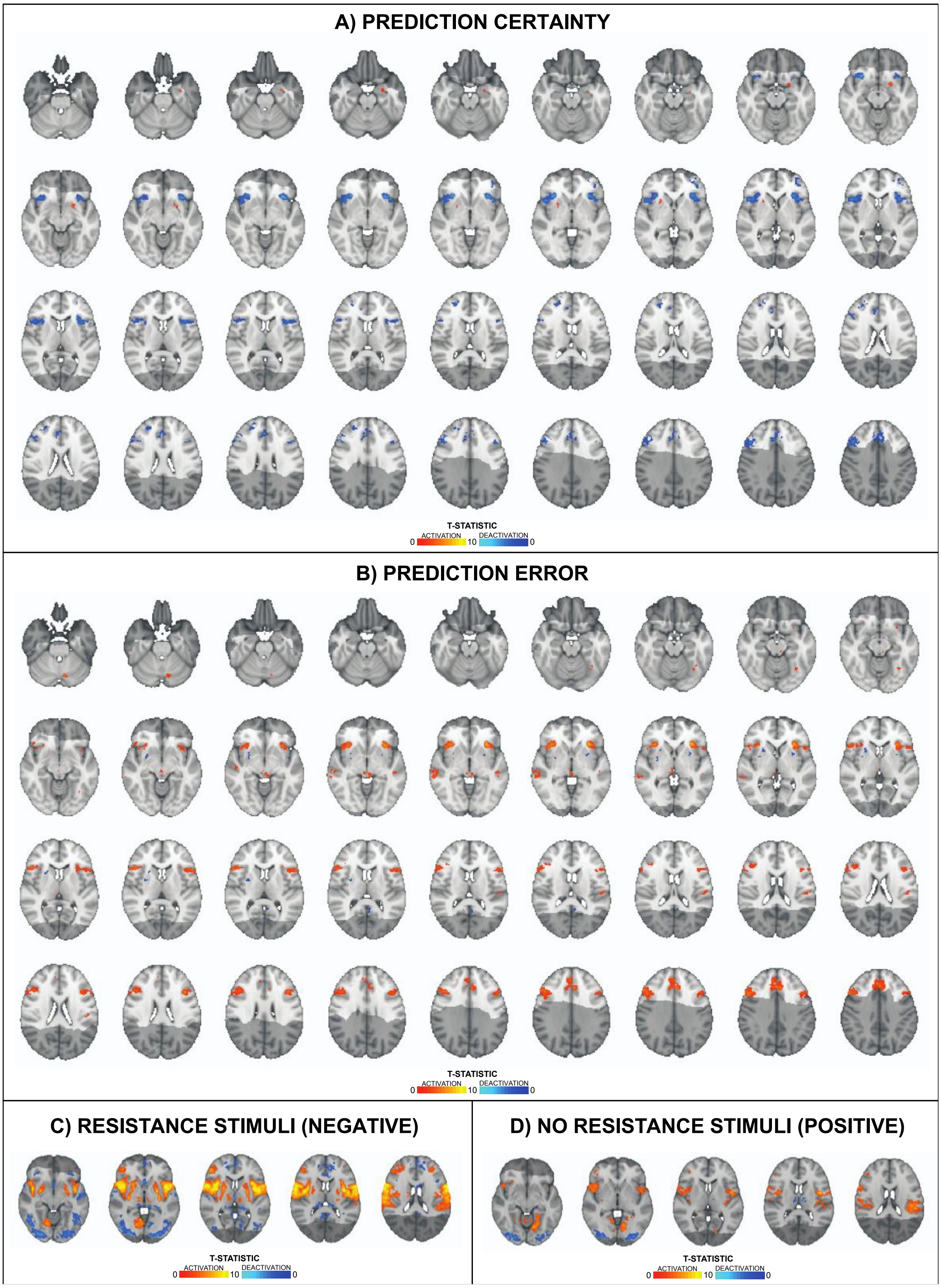

Supplementary Figure 6. BOLD activity related to prediction certainty, prediction errors, resistance and no-resistance stimuli, related to Figures 4 and 5. A) Significant BOLD activity associated with prediction certainty, averaged over positive and negative. B) Significant BOLD activity associated with prediction error magnitude, averaged over positive and negative. C) Significant BOLD activity associated with inspiratory resistance periods. D) Significant BOLD activity associated with no resistance periods. The images consist of a colour-rendered statistical map superimposed on a standard (MNI 1x1x1mm) brain. The bright grey region represents the coverage of the coronal-oblique functional scan. Significant regions are displayed with a cluster threshold of $p<0.05$, FWE corrected for multiple comparisons across all voxels included in the functional volume. Images in A and B are an expanded view of Figure 4.

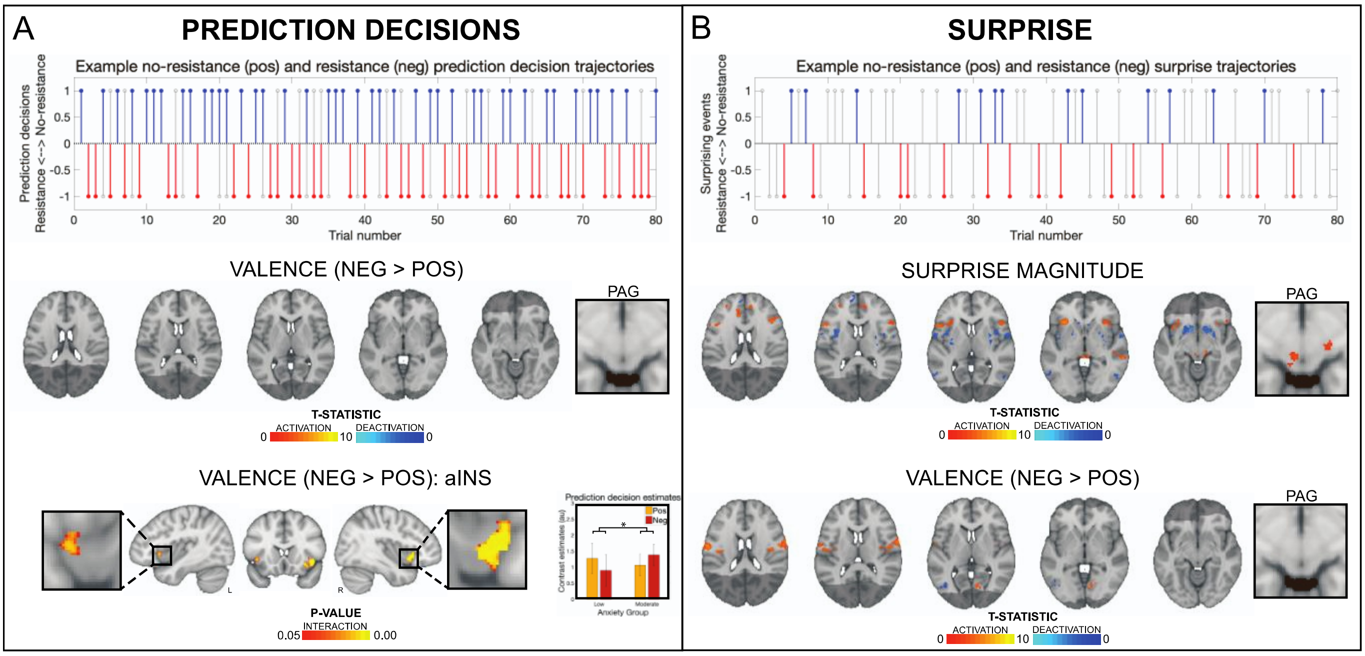

Supplementary Figure 7. Overall results from the non-computational decision-based analyses of the 'Breathing Learning Task' (BLT), related to Figures 4 and 5. The plots in both (A) and (B) demonstrate how prediction decisions (in A) and surprise (in B) trajectories are encoded into positive (i.e. towards no resistance) and negative (i.e. towards resistance, red) values. The grey lines in both plots represent the stimulus at each trial, while the blue (positive) and red (negative) lines in (A) denote the prediction decisions (prior to the stimulus) and in (B) the surprising events (where the prediction decision was incorrect). In both trajectories the dotted black line denotes the boundaries between positive and negative valence, and the distance from the dotted line is taken as the final value (i.e. absolute values). The brain images in (A) represent the influence of valence on prediction decisions (difference between negative and positive decisions), while there is no equivalent representation of overall predictions in a binary decision model compared to the computational model design (the average over positive and negative prediction decisions simply represents the cue presentation). The bottom panel of brain images in (A) demonstrate an interaction effect between valence (i.e. positive vs. negative) and anxiety group (low vs. moderate) for the anterior insula activity related to the valence of the prediction decisions of interoceptive breathing stimuli. Here, voxel-wise statistics were performed using non-parametric permutation testing within a mask of the anterior insula and periaqueductal gray, with significant results determined by p<0.05 (corrected for multiple comparisons within the mask). The brain images in (B) represent the activity associated with average surprise (average over positive and negative surprise trajectories) and the influence of valence on surprise (difference between negative and positive surprise trajectories). The images consist of a colour-rendered statistical map superimposed on a standard (MNI 1x1x1mm) brain. The bright grey region represents the coverage of the coronal-oblique functional scan. Significant regions are displayed with a cluster threshold of p<0.05, FWE corrected for multiple comparisons across all voxels included in the functional volume. Abbreviations: PAG, periaqueductal gray

Supplementary Table 1. Physiological summaries and group comparison results from the stimulus periods of the 'Breathing Learning Task' (BLT), related to Figure 3. Abbreviations: Wxn, Wilcoxon rank sum test; Ttest, students independent T-test. If a Wilcoxon rank sum test was utilised, reported values are median ± interquartile range.

	Total	Low	Moderate	P-value	Test
RESISTANCE					
70% of maximum inspiratory pressure	-58.8 (24.5)	-63.4 (23.8)	-56.0 (28.0)	0.52	Wxn
Avg measured pressure (cmH_2O)	-4.0 (3.7)	-4.2 (3.8)	-3.8 (2.9)	0.72	Wxn
Max measured pressure (cmH_2O)	-7.3 (4.9)	-7.3 (6.7)	-7.3 (3.9)	0.68	Wxn
Avg breathing rate (bpm)	13.6 (6.7)	14.6 (5.6)	13.0 (10.1)	0.39	Wxn
Avg breathing depth (% of rest depth)	103.9 (25.5)	97.0 (23.7)	105.9 (26.1)	0.29	Ttest
Heart rate (bpm)	66.3 (13.5)	66.9 (12.5)	66.0 (15.6)	0.69	Ttest
NO RESISTANCE					
Avg measured pressure (cmH_2O)	-0.1 (0.1)	-0.1 (0.1)	-0.1 (0.1)	0.05	Wxn
Max measured pressure (cmH_2O)	-0.8 (0.5)	-0.8 (0.8)	-0.7 (0.4)	0.59	Wxn
Avg breathing rate (bpm)	14.8 (5.1)	14.9 (5.0)	14.7 (5.8)	0.92	Ttest
Avg breathing depth (% of rest depth)	106.6 (17.0)	105.0 (17.0)	108.8 (21.7)	0.35	Ttest
Heart rate (bpm)	66.7 (12.3)	67.6 (12.1)	66.6 (14.0)	0.84	Wxn

Supplementary Table 2. Parameter configurations and priors for each of the candidate models, related to Figure 3. If the prior variance is set to 0 for a parameter it is not estimated, and the prior mean and variance for the estimated parameters (in bold) were taken from the maximum likelihood fits of the pilot participant data. Prior means are given in native space, prior variances in estimation (transformed) space.

Rescorla Wagner			
Parameter	Prior mean	Prior variance	Transformation
$v^{(0)}$	0.5	0	logit
**α**	**0.29**	**2.54**	**logit**
**Observation model ζ**	**2.14**	**3.33**	**log**
Hierarchical Gaussian Filter (2-level)			
Parameter	Prior mean	Prior variance	Transformation
$\mu_2^{(0)}$	0	0	none
$\mu_3^{(0)}$	1	0	none
$\sigma_2^{(0)}$	0.1	0	log
$\sigma_3^{(0)}$	1	0	log
ρ_2	0	0	none
ρ_3	0	0	none
κ_1	1	0	log
κ_2	0	0	log
**ω_2**	**-0.70**	**1.61**	**none**
ω_3	$-\infty$	0	none
**Observation model ζ**	**2.14**	**1.17**	**log**
Hierarchical Gaussian Filter (3-level)			
Parameter	Prior mean	Prior variance	Transformation
$\mu_2^{(0)}$	0	0	none
$\mu_3^{(0)}$	1	0	none
$\sigma_2^{(0)}$	0.1	0	log
$\sigma_3^{(0)}$	1	0	log
ρ_2	0	0	none
ρ_3	0	0	none
κ_1	1	0	log
**κ_2**	**2.39**	**0.30**	**log**
ω_2	-3	0	none
ω_3	-6	0	none
**Observation model ζ**	**2.14**	**1.21**	**log**

Supplementary Table 3. Parameter recovery metrics for each of the candidate models, related to Figure 3. R values are Pearson Correlation Coefficients that have been Fisher's Z-transformed prior to averaging across the 10 simulation runs, and then back-transformed into R values. Each simulation run consisted of 60 simulated synthetic datasets sampled from the prior distribution of values for the perceptual model, repeated at each noise level ($\zeta = [1,5,10]$), and recovered using MAP estimation.

Rescorla Wagner						
	$\zeta = 1$		$\zeta = 5$		$\zeta = 10$	
	Mean	Std	Mean	Std	Mean	Std
α R	0.88	0.07	0.96	0.14	0.97	0.11
ζ_{est}	1.08	1.70	5.82	1.56	10.62	1.61
Hierarchical Gaussian Filter (2-level)						
	$\zeta = 1$		$\zeta = 5$		$\zeta = 10$	
	Mean	Std	Mean	Std	Mean	Std
ω_2 R	0.83	0.10	0.95	0.14	0.96	0.15
ζ_{est}	1.11	1.47	5.05	1.36	7.89	1.57
Hierarchical Gaussian Filter (3-level)						
	$\zeta = 1$		$\zeta = 5$		$\zeta = 10$	
	Mean	Std	Mean	Std	Mean	Std
κ_2 R	0.32	0.31	0.49	0.49	0.56	0.59
ζ_{est}	1.01	1.35	4.92	1.50	8.52	1.75

Supplementary Table 4. Whole and group-wise model comparison results, related to Figure 3. Abbreviations: XP, exceedance probability; PXP, protected exceedance probability.

	RW	HGF2	HGF3
Whole group			
XP	0.01	0.99	0.00
PXP	0.30	0.40	0.30
Low anxiety			
XP	0.30	0.24	0.46
PXP	0.33	0.33	0.34
Moderate anxiety			
XP	0.01	0.99	0.00
PXP	0.26	0.48	0.26

Supplementary Table 5. Behavioural group comparison results from the 'Breathing Learning Task' (BLT) with all participants included, related to Table 1. Fitted perceptual and response model parameters are learning rate (α) and inverse decision temperature (ζ). Abbreviations: Wxn, Wilcoxon rank sum test; Ttest, students independent T-test. If a Wilcoxon rank sum test was utilised, reported values are median ± interquartile range.

Learning rate (α)	0.25 (0.18)	0.24 (0.14)	0.25 (0.21)	0.64	Wxn
Inv. decision temp (ζ)	2.60 (3.42)	2.70 (3.12)	2.32 (3.65)	0.88	Wxn

*Supplementary Table 6. Exploratory regression analysis conducted on the fitted model learning rate parameter and the subjective ratings of breathing difficulty and anxiety, related to Figure 3. Regression parameters consisted of trait anxiety scores (from the STAI-T questionnaire), depression scores (from the CES-D questionnaire) and gender (male=1). **Significant coefficient at p<0.05 with multiple comparison correction for the three exploratory regression models.*

	Trait anxiety	p-value	Depression score	p-value	Gender (male)	p-value
Learning rate	< 0.01	0.99	< 0.01	0.50	**-0.14**	**< 0.01****
Breathing anxiety	1.44	0.02	0.16	0.85	-1.77	0.77
Breathing difficulty	0.59	0.05	-0.74	0.08	5.49	0.08

Supplementary Table 7. Correlation matrix across task modalities, with Pearson's (A) and Spearman's (B) correlation coefficients given above the diagonal and p values below the diagonal, related to Figure 6. Correlations with a p value<0.05 are represented in bold text, and those p<0.01 are shaded grey. Variables: 1) State anxiety (STAI-S); 2) Anxiety disorder (GAD-7); 3) Anxiety sensitivity (ASI); 4) Depression (CES-D); 5) Body perception (BPQ); 6) Interoceptive awareness (MAIA); 7) Breathing-related catastrophising (PCS-B); 8) Breathing-related vigilance (PVQ-B); 9) Perceptual threshold (from the FDT); 10) Decision bias (from the FDT); 11) Metacognitive bias (average confidence, from the FDT); 12) Metacognitive performance (from the FDT); 13) BOLD activity associated with positive predictions (from the BLT); 14) BOLD activity associated with negative predictions (from the BLT); 15) BOLD activity associated with positive prediction errors (from the BLT); 16) BOLD activity associated with negative prediction errors (from the BLT).

A) CORRELATION MATRIX: PEARSON

	1	2	3	4	5	6	7	8	9	10	11	12	13	14	15	16
1		**0.70**	**0.57**	**0.72**	0.22	**-0.57**	**0.42**	0.06	**0.26**	**-0.28**	**-0.30**	-0.23	-0.04	0.10	0.01	-0.10
2	**<0.01**		**0.55**	**0.72**	**0.35**	**-0.51**	**0.39**	0.12	0.18	-0.16	-0.25	-0.17	-0.07	0.04	0.12	-0.15
3	**<0.01**	**<0.01**		**0.68**	**0.40**	**-0.40**	**0.62**	0.20	0.20	-0.18	**-0.30**	-0.21	0.01	0.13	0.16	-0.03
4	**<0.01**	**<0.01**	**<0.01**		**0.31**	**-0.43**	**0.61**	**0.27**	**0.28**	-0.14	-0.24	-0.17	-0.01	0.13	0.12	-0.10
5	0.10	**0.01**	**<0.01**	**0.02**		-0.16	0.25	-0.02	0.04	0.19	-0.03	-0.12	0.06	0.06	0.06	-0.08
6	**<0.01**	**<0.01**	**<0.01**	**<0.01**	0.24		**-0.34**	0.02	-0.13	0.07	0.23	0.16	0.14	-0.01	-0.12	0.07
7	**<0.01**	**<0.01**	**<0.01**	**<0.01**	0.05	**0.01**		**0.49**	-0.01	0.01	-0.21	**-0.30**	0.17	0.24	-0.05	-0.10
8	0.66	0.36	0.13	**0.04**	0.89	0.87	**<0.01**		-0.10	-0.05	0.10	0.06	0.16	0.13	0.09	-0.08
9	**0.05**	0.17	0.13	**0.03**	0.78	0.34	0.97	0.48		0.20	0.05	0.02	-0.12	-0.03	0.03	-0.10
10	**0.04**	0.23	0.18	0.29	0.16	0.62	0.95	0.69	0.13		**0.35**	0.02	-0.06	0.01	0.14	0.06
11	**0.02**	0.06	**0.02**	0.07	0.85	0.08	0.12	0.46	0.70	**0.01**		**0.35**	-0.21	**-0.28**	-0.00	0.07
12	0.09	0.20	0.11	0.20	0.38	0.22	**0.02**	0.65	0.87	0.87	**0.01**		-0.23	-0.23	0.13	**0.42**
13	0.75	0.60	0.91	0.96	0.64	0.29	0.19	0.22	0.36	0.68	0.12	0.08		**0.79**	-0.22	**-0.30**
14	0.44	0.76	0.31	0.34	0.68	0.97	0.07	0.32	0.83	0.94	**0.03**	0.08	**<0.01**		-0.10	**-0.46**
15	0.92	0.39	0.23	0.37	0.64	0.36	0.72	0.52	0.80	0.28	0.98	0.33	0.10	0.45		0.08
16	0.45	0.26	0.84	0.46	0.53	0.58	0.46	0.57	0.45	0.63	0.60	**<0.01**	**0.02**	**<0.01**	0.57	

B) CORRELATION MATRIX: SPEARMAN

	1	2	3	4	5	6	7	8	9	10	11	12	13	14	15	16
1		**0.71**	**0.55**	**0.77**	**0.26**	**-0.57**	**0.34**	0.05	0.24	-0.18	-0.22	-0.23	-0.18	0.06	0.07	-0.03
2	**<0.01**		**0.53**	**0.67**	**0.40**	**-0.51**	**0.27**	0.10	0.13	-0.18	**-0.27**	-0.13	-0.12	0.01	0.15	-0.05
3	**<0.01**	**<0.01**		**0.67**	**0.47**	**-0.36**	**0.58**	0.22	0.19	-0.15	**-0.29**	-0.14	-0.10	0.13	0.22	0.04
4	**<0.01**	**<0.01**	**<0.01**		**0.37**	**-0.40**	**0.51**	0.19	0.24	-0.22	**-0.26**	-0.13	-0.07	0.11	0.21	0.01
5	**0.05**	**<0.01**	**<0.01**	**<0.01**		-0.17	**0.26**	0.03	0.05	0.08	-0.05	-0.01	-0.01	0.01	0.20	0.01
6	**<0.01**	**<0.01**	**0.01**	**<0.01**	0.20		**-0.28**	-0.00	-0.11	0.04	0.15	0.08	0.03	-0.07	-0.15	0.03
7	**0.01**	**0.04**	**<0.01**	**<0.01**	**0.05**	**0.04**		**0.56**	-0.01	0.03	-0.24	**-0.29**	0.17	0.22	-0.06	0.02
8	0.71	0.47	0.10	0.16	0.82	0.98	**<0.01**		-0.08	-0.11	0.03	-0.04	0.17	0.09	0.10	-0.06
9	0.07	0.35	0.16	0.07	0.73	0.42	0.96	0.54		0.14	0.13	0.02	-0.10	0.06	-0.03	-0.14
10	0.18	0.17	0.26	0.10	0.57	0.79	0.82	0.40	0.30		**0.34**	0.07	-0.08	-0.10	0.07	0.12
11	0.10	**0.04**	**0.03**	**0.04**	0.68	0.27	0.07	0.81	0.34	**0.01**		0.32	-0.10	-0.18	-0.00	0.07
12	0.08	0.32	0.31	0.31	0.92	0.55	**0.03**	0.77	0.90	0.59	**0.01**		-0.20	-0.23	**0.30**	**0.47**
13	0.18	0.38	0.45	0.63	0.92	0.84	0.19	0.20	0.44	0.55	0.47	0.14		**0.62**	-0.21	-0.08
14	0.67	0.92	0.33	0.42	0.97	0.60	0.10	0.51	0.67	0.47	0.18	0.08	**<0.01**		-0.19	**-0.32**
15	0.59	0.25	0.09	0.12	0.13	0.27	0.66	0.44	0.81	0.63	0.98	**0.02**	0.11	0.16		0.20
16	0.82	0.69	0.78	0.95	0.97	0.84	0.89	0.66	0.31	0.37	0.62	**<0.01**	0.54	**0.01**	0.12	
